# General Medicine and Therapeutics

**Published:** 1895-12

**Authors:** 


### *SELECTIONS AND ABSTRACTS.*

---

### GENERAL MEDICINE AND THERAPEUTICS.

Edited by Dr. Luther B. Grandy.

### Malarial Hematuria.

Dr. Jones, of Austin, Texas, contributes a paper to the *Texas Medical Journal* for September, on "Hemorrhagic Malarial Fever," which covers the ground very well. Following is an abstract:

The disease is widely disseminated in the Southern States, but is not always coexistent with other forms of malaria, I having known a number of localities where intermittents were quite prevalent and this disease quite unknown. Neither is it absolutely confined to malarial localities, according to some writers cases having occurred in localities immune from all other malarial manifestations.

Hemorrhagic malarial fever is an acute disease, characterized by rigors and icteric hue of the skin and all the tissues of the body; the discharge of dark-colored, bloody urine; intense nausea and nervous prostration, and usually, though not invariably, with fever.

It does not appear to be self-limited, and the tendency is to death, unless checked by appropriate treatment. The duration is from a few hours to ten or twelve days. In some cases there has been observed a tendency to recur on the seventh and fourteenth days.

I have carefully analyzed over four hundred cases of the disease, as reported from various localities, and from these have selected sixty-seven that fully represent its salient features. These sixty-seven cases are used in preference to the others simply because they were fully reported.

Of these sixty-seven cases, thirty-nine recovered and twenty-eight died, a mortality of 48.80 per cent. In fifty-one cases the disease was ushered in by a chill; in nine without the chill; in seven cases chill not mentioned; in thirty-two cases the initial chill was the only one experienced; twelve cases had no chills; six cases more than two; seven cases no mention. Nausea and vomiting occurred in fifty-six cases; four cases no nausea and vomiting; seven cases no mention. Thirty-nine cases had fever over 101°; ten did not; eighteen not reported. All of the sixty-seven had bloody urine, with remission of this symptom in four cases and complete intermission in four. Sixty-four are reported as jaundiced; no mention of jaundice in three cases. There were six cases with some hemorrhage from stomach, though in only one case was it compared to the black vomit of yellow fever. One case had hemorrhage from bowels and lungs.

It will be seen by this table that the average age was twenty-five. Half the entire number were between ten and thirty, three-fourths between ten and forty, showing that the disease attacks preferably those in the prime of life. Almost twice as many males as females were attacked. While the proportion of whites to blacks is twenty to one, although the blacks outnumber the whites largely in these districts.

A man in the prime of life, who has been having intermittents, possibly for several months, is seized with a severe chill, or more properly rigor, for it resembles a surgical rigor more than a malarial chill. As soon as the chill passes off, or before, the patient passes a pint or more of very dark, bloody-looking urine, which, if dropped on a piece of white paper and allowed to dry, will make a deep yellow stain. *There are no clots or bright-looking blood.*

There may be fever following chill, but it is not usually high, and, indeed, many of the severer cases run their course with a subnormal temperature. A moderately hight temperature is favorable. The skin soon becomes jaundiced, varying in shade from a lemon to a deep bronze. Nausea of the most intense character is soon established, frequently accompanied by projectile vomiting. Thirst is intense. The bowels are constipated, and the stools, whether procured by mercurials or other means, are dark green and tarry. The fluids are all tinctured with bile. Many have reported that when blisters were resorted to, the vesicles filled with blood. I am persuaded that this is a mistake, as I have invariably blistered my patients and have found that the serum was of a brownish color from bile, but have seen no blood. In fact, there is no general tendency to hemorrhage. The patient may be delirious; if so, it will be low muttering or wild excitement. The skin is very dry and hard. The urine is loaded with the material of disintegrated red-blood corpuscles, epithelium from the kidney, mucus, bile, some fresh blood, and albumin usually in large quantity. If the attack is of severe type, the secretion of urine begins to diminish, and unless the flow can be stimulated, will soon be entirely suppressed. Any considerable suppression means death. Of the twenty-seven cases terminated, twenty-one cases of suppression occurred. Of these eighteen died; the suppression in the other three was reported to be "slight." The patient usually has but one chill, and that is at the beginning, but this is not invariable, some cases having periodical chills, with exacerbations and remissions of all the symptoms. These cases usually terminate favorably. Rigors occur at frequent intervals in severe cases, accompanied by suppression of the urine. There is but little pain complained of, and that is referred to the lumbar region. A few cases have severe colic during convalescence. The case may terminate in death or recovery in a few hours, or may continue for ten or twelve days. If an unfavorable termination can be warded off until the seventh day is passed, recovery is the rule.

It is a mooted question whether the jaundice, which is such a constant symptom, is of hematogenous or hepatogenous origin. Tyson, Hare, and Beranger-Ferrand favor the former, and Joseph Jones the latter view. I am inclined to think that any one who is familiar with the disease, clinically, and observes the flood of bile pouring from the kidneys and intestines, staining every tissue of the body, and even in some cases escaping through the pores of the skin, will not believe that it is all derived from the broken down blood discs. The breaking down of the corpuscles occurs in the circulation and not in the bladder, as conclusively demonstrated by Sternberg and Martin.

One more curious clinical fact, and I am done with this branch of the subject. It almost invariably occurs that at the end of the first chill the patient voids a pint or more of bloody urine. This amount of urine could not possibly have been secreted in this short time, hence the chill could not have caused the bloody urine; and as I have remarked above, the chill more nearly resembles a surgical rigor than the cold stage of an intermittent; hence, it is my belief that the *bloody urine causes the chill or rigor.*

As to treatment, I will say but little. Quinine has been regarded as specific; but a glance at the accompanying table shows that it is equally powerless to prevent or arrest the disease. In fact, owing to the hyperemia and inflammation of the kidneys, it is clearly contraindicated. It is also contraindicated on account of its well known effect of lessening the oxygen-carrying power of the red-blood corpuscles. As these are the point of attack in this disease, and are so deficient in number, nothing should be done to further impair their efficiency. Indeed, Hare, Kitasato, Martin of Mississippi, and many others report cases where quinine has certainly caused the disease, or at least precipitated it, and I have seen it produce a relapse in cases that were convalescent. Fortunately for the patients treated with this drug, their stomachs are usually so rebellious that it is impossible to cinchonize them usually. It will be seen in the subjoined table of cases that of the sixty-seven cases tabulated, no case died where purgatives were given and acted the first day. Purgatives were given with effect in forty-three cases; of these only ten died, a mortality of 23.3 per cent. Given without effect in six cases, with five deaths; mortality, 83 per cent. Purgatives were not given in eleven cases, with eight deaths; mortality, 72.7 per cent. Purgatives were given—effects not stated—in three cases, with two deaths; mortality, 66⅔ per cent. Purgatives not reported on in six cases, with four deaths; mortality, 80 per cent.; hence, it will be seen that purgation is highly beneficial. This can be best secured by small doses (one or two grains) of *English* calomel every hour. This should be aided by copious enemas of normal saline solution (Dr. J. B. Pease, Miss.), repeated at least every two or three hours. This, besides aiding the action of the mercurial, stimulates the failing heart by replacing the liquor sanguinis that is being constantly lost, and encourages free diaphoresis, which materially relieves the overworked kidneys. Strychnia should be given hypodermically from the outset, and in doses sufficient to produce its physiological effect. Dr. J. J. Onendorf, of

Sharkey county, Mississippi, assures me that he has given it in doses of one-quarter grain hypodermically every six hours with good effect. When kidneys are failing, give turpentine freely. I have always used a cantharidal blister over stomach and liver, and believe that it relieves the distressing nausea, and by relieving the congestion of the liver, aids in restoring its normal action. Theoretically jaborandi, or its active principle, should be useful in relieving the overworked kidneys, but should be given guardedly. The disease being peculiarly adynamic, supportive measures are of the utmost importance; but owing to the unfortunate condition of the stomach but little food can be given, beef tea, highly seasoned with red pepper, being the most useful in my experience. As the patient convalesces perfect quietude must be enforced, as I have known a number of patients to die suddenly on slight exertion after convalescence was thoroughly established.

---

### ANALGESIAAND SEDATION.—AN ESSENTIAL ADJUNCT TO TREATMENT.

In a late article under this heading, Dr. John J. Sullivan, of New York, says that on account of the frequency with which pneumonia in late years is accompanied with grippal symptoms, the treatment, to a great extent, has been modified or changed. The essential features in the result desired are a diminution of the pain and a lowering of the temperature. Opinions differ as to whether a reduction of the temperature influences the course of the disease, but a consensus of opinion is that antipyretic treatment is distinctly called for in the beginning, and an analgesic at all times, if needed to assuage suffering. The antipyretic should be Antikamnia, and the analgesic is supplied by Codeine and Antikamnia together. This is given every three or four hours in tablets containing $4\frac{3}{4}$ grains Antikamnia and $\frac{1}{4}$ grain Codeine, throughout the period of congestion and consolidation. Where there is great restlessness, this will have a delightful effect.

In the nocturnal pains of syphilis, in the grinding pains which precede labor, and the uterine contractions which often lead to abortion in tic-douloureux, brachialgia, cardialgia, gastralgia, hepatalgia, nephralgia, and dysmenorrhea, immediate relief is afforded by the use of this combination, and the relief is not merely temporary and palliative, but in very many cases curative.

In the neuroses of the respiratory organs, great relief is afforded by the use of this combination. A paroxysm of asthma is often cut short by a full dose; hay-fever or autumnal catarrh is benefited by its use.

In the harassing cough of phthisis, or in the pain of pleuritis, in the painful sensations accompanying bronchitis when the tubes are dry and irritable—as they usually are—the blending of Codeine and Antikamnia will not be found wanting in its action, but will give results that are gratifying to both the patient and the medical attendant. As a producer of sleep, it will be found efficacious This is doubly true when there is great nervous excitement.

In pulmonary diseases this combination is worthy of trial. It is a sedative to the respiratory centers in both acute and chronic disorders of the lungs. Cough in the vast majority of cases is promptly and lastingly decreased and often entirely suppressed. In diseases of the respiratory organs, pain and cough are the symptoms which especially call for something to relieve; this tablet does it, and in addition controls the violent movements accompanying the cough, and which are so distressing.

This combination is the remedy for diabetes and is superior to any other in diminishing the quantity of sugar in the urine, and also in diminishing the quantity of urine itself in diabetes mellitus. The bulimia and polydipsia are lessened by its use, and probably the changes in the nervous system, which accompany or are causative of the disease, are arrested or prevented. It also prevents waste. It controls restlessness; it relieves insomnia; it relieves distressing nervous symptoms. It relieves the craving of the stomach, and lessens the frequency of the calls to urinate.

It is not claimed that the combination will cure diabetes mellitus, but there will be, in many cases, arrest of the disease, with prolonged periods of good health, and cure in some cases.

### TABLE OF WEIGHTS, HEIGHTS, ETC.

The following, taken from the Boston *Journal of Health,* may be taken as the standard of weight and measurements for a well developed male adult :

| HEIGHT. | Weight. | Chest. | Waist. |
|---|---|---|---|
| 5 feet | 103–107 | 32–33 | 29 |
| 5 feet 1 inch | 107–111 | 33–34 | 29½ |
| 5 feet 2 inches | 111–116 | 34–35 | 30 |
| 5 feet 3 inches | 116–121 | 35–36 | 30½ |
| 5 feet 4 inches | 121–127 | 36–37 | 31 |
| 5 feet 5 inches | 127–133 | 37–38 | 31½ |
| 5 feet 6 inches | 133–140 | 38–39 | 32 |
| 5 feet 7 inches | 140–147 | 39–40 | 32½ |
| 5 feet 8 inches | 147–155 | 40–41 | 33 |
| 5 feet 9 inches | 155–164 | 41–42 | 33½ |
| 5 feet 10 inches | 164–174 | 42–43 | 34 |
| 5 feet 11 inches | 174–185 | 43–44 | 34½ |
| 6 feet | 185–196 | 44–45 | 35 |

---

### NEURASTHENIC HEADACHE.

According to Dr. Allan McLane Hamilton headaches are quite various as well as varied. The "neurasthenic" group includes a long list of irregular headaches of obscure origin, but dependent more or less upon conditions of neural weakness; many are due to eye strain, many to ovarian or uterine disorders, but in most cases no cause can be discovered. So recourse must be had to general measures. The bromide of caffeine is often serviceable. The following formula is recommended :

℞ Ammonii carbonatis ...... ℨ iij.

Tinct. moschi ...... ℨ vj.

Spts. lavandulæ ...... ℥ j.

Elix. ammonii valerianatis ...... ℥ viij.

M. Sig.—Two teaspoonfuls at a dose in water.

The most efficacious preparations for continuous treatment are those of the restorative class. A pill of arseniate of strychnine, strophanthus, and quinine may be tried with expectation of great benefit, thus:

℞ Strychninæ arseniat ........................................ gr. ss.

Sem. strophanth ........................................ gr. vj.

Quininæ sulphat ........................................ ℈ ijss.

M. Ft. pilulæ no. xlviij.

Sig.—One or two after each meal.

Ethereal acetate of iron may be administered one hour after the meal with great advantage in many cases.—*Medical Record.*

